# Crocetin promotes angiogenesis in human endothelial cells through PI3K-Akt-eNOS signaling pathway

**DOI:** 10.17179/excli2019-1175

**Published:** 2019-10-21

**Authors:** Mahdieh Nasirzadeh, Yousef Rasmi, Reza Rahbarghazi, Fatemeh Kheradmand, Mojtaba Karimipour, Pornanong Aramwit, Maryam Astinfeshan, Zafar Gholinejad, Behrokh Daeihasani, Ehsan Saboory, Alireza Shirpoor, Aysa Rezabakhsh, Elmira Zolali, Naser Khalaji

**Affiliations:** 1 Department of Biochemistry, Faculty of Medicine, Urmia University of Medical Sciences, Urmia, Iran; 2 Cellular and Molecular Research Center, Urmia University of Medical Sciences, Urmia, Iran; 3 Department of Applied Cell Sciences, Faculty of Advanced Medical Sciences, Tabriz University of Medical Sciences, Tabriz, Iran; 4 Department of Anatomy, Faculty of Medicine, Urmia University of Medical Sciences, Urmia, Iran; 5 Department of Pharmacy Practice, Faculty of Pharmaceutical Sciences, Chulalongkorn University, Phaya Thai Road, Phatumwan, Bangkok 10330, Thailand; 6 Department of Biology, Payame Noor University, P.O.Box 19395-3697, Tehran, Iran; 7 Neuroscience Research Center, Faculty of Medicine, Urmia University of Medical Sciences, Urmia, Iran; 8 Department of Physiology, Faculty of Medicine, Urmia University of Medical Sciences, Urmia, Iran; 9 Aging Research Institute, Tabriz University of Medical Sciences, Tabriz, Iran; 10 Department of Pharmacology and Toxicology, Faculty of Pharmacy, Tabriz University of Medical Sciences, Tabriz, Iran

**Keywords:** crocetin, human endothelial cells, angiogenesis, tube formation, migration, VEGFR-2- Akt-eNOS signaling

## Abstract

Previous studies proved the pro-angiogenic effect of Crocetin, a natural carotenoid dicarboxylic acid, in both *in vivo* and *in vitro* models. However, the exact mechanism of Crocetin action has not completely been elucidated yet. The current experiment was designed to find the activity of PI3K-Akt-eNOS axis after the treatment of endothelial cells with Crocetin *in vitro*. Human Umbilical Vein Endothelial Cells (HUVECs) were incubated with various concentrations of Crocetin (1, 5, 25, 50, and 100 µM) over a period of 72 h. Crocetin significantly increased HUVECs viability after 72 h as compared with the control group. We also found that Crocetin promoted the formation of the capillary-like structure compared to the control (*p*<0.05). Moreover, an improved migration rate and increased MMP-9 activity were observed in HUVECs that received 50 µM Crocetin (*p*<0.05). Crocetin enhanced the uptake of Ac-LDL which is correlated with increased lipid metabolism. Based on the data from the current experiment, protein level of VEGFR-1, -2 and p-Akt/Akt, p-eNOS/eNOS ratios were increased 72 h after the treatment of HUVECs with Crocetin (*p*<0.05). In contrast, the transcription level of VEGF was reduced in Crocetin-treated cells. These data demonstrated that Crocetin promotes HUVECs angiogenesis potential by the modulation of VEGF signaling pathway and increased cell viability. The PI3K/Akt/eNOS axis is required for a Crocetin-associated activity in endothelial cells.

## Introduction

Angiogenesis, the formation of new blood vessels from the pre-existing vascular bed, is a complex multistep and tightly controlled process. This phenomenon commonly occurs in both physiological and pathological conditions by the help of different pro- and anti-angiogenic factors (Folkman, 1971[9], 1995[8]). Different sequential steps are involved to promote endothelial angiogenic response. It was previously stated that the increase of blood circulation is an essential response to limit the extent of the lesion at the site of hypoxic or ischemic tissues (Battegay, 1995[1]). Vascular endothelial growth factor (VEGF), termed as an endothelial lineage-specific mitogen, is produced by different cell types at the proximity to endothelial cells (ECs) to increase angiogenesis. This factor has been used as a therapeutic drug for the alleviation of several human diseases, such as cerebral ischemic injury, myocardial infarction, limb ischemia, and numerous wounds (Isner and Asahara, 1999[12]). Any disturbance in the dynamic of VEGF and single gene mutation in VEGF allele contributes to abnormal angiogenesis rate and embryonic lethality. For successful induction of angiogenic signaling pathway, the interaction of ligand VEGF with ECs surface receptor VEGFR-2 is required. Upon the interaction of VEGFR-2 with VEGF, VEGFR-2 cytoplasmic domain is activated, leading to the activation of multiple downstream effectors, including PI3K/Akt and eNOS signaling pathway (Chen et al., 2007[3]). Therefore, identification of novel pro-angiogenic bioactive compounds appears to be the primary target for regenerative medicine.

**Figure 1:**
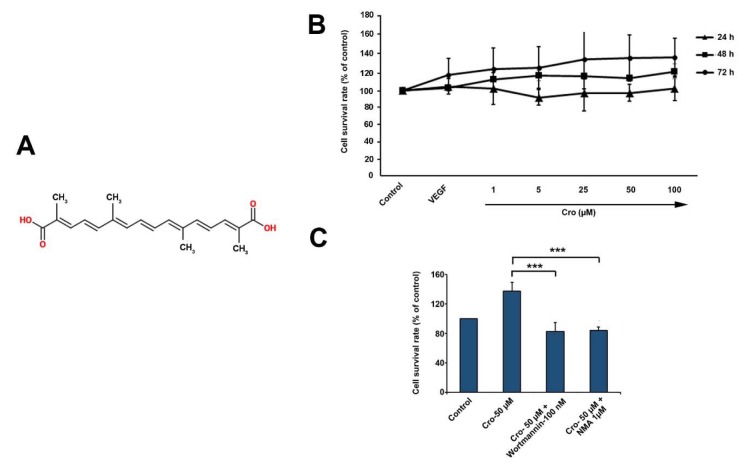
The molecular formula of Crocetin (A). Relative viability measured by WST-1 in HUVECs exposed to various concentrations of Crocetin over a period of 72 h (B) (n=12). Measuring HUVECs viability in the combined regime of Crocetin, Wortmannin (a PI3K inhibitor) and NMA (an eNOS inhibitor) (C). Data are expressed as mean ± SD (n=12). *p<0.05, **p<0.01, ***p<0.001 using one-way ANOVA with Tukey post-hoc test.

So far, great bodies of studies have discovered the pro-angiogenic activity of diverse phyto-compounds through engaging multiple signal pathways inside ECs (Chung et al., 2008[5], 2010[6]; Kang et al., 2013[13]). Among these compounds, Crocetin is commonly found in *Crocus sativus* (saffron). Saffron, which is a perennial herb, belongs to the family of Iridaceae and has various biological effects. Previously, it was reported that Crocetin, 8,8´-diapocarotenedioic acid, 2,6,11,15-tetramethylhexadeca-2,4,6,8,10,12,14-heptaenedoic acid, possesses multi-unsaturated conjugate olefine acid structure (Figure 1A(Fig. 1)). It was reported that Crocetin possesses antioxidative, hepatoprotective, neuroprotective, anti-inflammatory and anti-atherosclerotic properties (Bie et al., 2011[2]; Song et al., 2016[18]; Umigai et al., 2012[19]; Yang et al., 2012[24]). To the best of our knowledge, different underlying mechanisms for pro-angiogenic signaling pathways have not yet been fully elucidated after exposure to Crocetin.

In the current experiment, we aimed to determine whether Crocetin can stimulate/inhibit angiogenesis in human ECs. The potential effect of Crocetin was also studied on VEGFR-2 signaling pathway by monitoring the PI3K/Akt and eNOS pathway. Results of the current experiment show the critical role of distinct molecular mechanisms in ECs after treatment with Crocetin.

## Materials and Methods

### Cell culture protocol

In the current study, we used Human Umbilical Vein Endothelial Cells (HUVECs, NCBI code: C554), purchased from Iranian National Cell Bank (Pasteur Institute, Iran). The cells were expanded in Dulbecco's Modified Eagle Medium: Nutrient Mixture F-12 (DMEM/F12, Biowest) enriched with 10 % fetal bovine serum (FBS, Biowest) and 1 % penicillin-streptomycin (Biowest). Following initial cell-plating density, culture flasks were transferred into a CO_2_ incubator with a humidified atmosphere at 37 °C. Cells between passages 3 to 6 were used for various analysis. For cell passage, HUVECs were detached using 0.25 % Trypsin-EDTA solution (Biowest).

### Treatment protocol

To address the possible pro- and/or anti-angiogenic effect, we exposed HUVECs to various concentrations of Crocetin (CAS no: 02193543; MP BIOMEDICALS), including 1, 5, 25, 50, and 100 µM for 24, 48 and 72 h. We prepared Crocetin stock solution in dimethyl sulfoxide (Merck) and stored in -20 °C until use. The final concentration of solvent was below 0.1 %.

### Cell viability assay

The cell survival rate was measured by using Premix WST-1 Cell Proliferation Assay (Cat no: MK400, TAKARA). In short, 200 µl of medium containing 1 × 10^4^ cells were filled in each well of 96-well plates (SPL) and were allowed to attach overnight. Prior to the treatment, we starved the cells by incubating in FBS-free medium for 1 h and incubated them with different Crocetin concentrations. Upon cell treatment at different time periods, the supernatant was discarded, replaced with 100 µl of Crocetin-free medium and followed by the addition of a WST-1 solution (10 µl per each well). The plates were kept in incubators for 2 h and the final optical density measured using a microplate ELISA reader (BioTek, USA) at 490 nm. The experiments were repeated in separate triplicate and the results were expressed as % of non-treated control. Cells receiving VEGF (Dilution: 10 ng/ml; Cat no: 279-85-15, Atocel) were used as a positive control. Based on the results from cell survival assay, we selected doses of 1 and 50 µM Crocetin for subsequent analysis.

### In vitro tube formation assay

After incubation for 72 hours with Crocetin, we performed *in vitro* tube formation assay in 48-well plates coated with 100 µl of growth factor-reduced Matrigel (Cat no: 356230; Corning). For this purpose, 500 µl medium containing 5 × 10^4^ cells, 1 % FBS, 1 or 50 mM Crocetin was transferred into each well and maintained for 8-24 hours. The average tubular number in 5 serial microscopic fields (HPF) and the average of tube length (µm) per total field area in 5 serial microscopic fields were also measured using AxioVision LE software version 4.8.2.0 (Carl Zeiss). In the control group, HUVECs were plated on Matrigel substrate in Crocetin and VEGF free condition. For positive control, cells were treated with 10 ng/ml VEGF. This assay was performed in triplicate. 

### Scratch assay

HUVECs were seeded at an initial density of 3×10^5^ cells per well in 6-well plates pre-coated with type I collagen. Confluent monolayers of HUVECs were washed twice with pre-warmed phosphate-buffered saline (PBS) solution and incubated with serum-free medium for 1-2 h at 37 °C. Thereafter, the monolayers of HUVECs were scratched in a straight line with a sterile pipette tip. After 72-h incubation, the distance between the edges was measured using AxioVision LE software in five random fields. Distance between scratch edges was calculated by the following formula; D_1_-D_2_ where D_1_ and D_2_ showed the distance between the edges at initial and respective time points, respectively.

### Transwell migration assay 

Cell migration assay was done using 24-well polycarbonate membrane cell culture plate with an 8-μm pore size (Cat No: 35224, SPL) inserts. 72 h after HUVECs exposure to Crocetin, 2×10^4^ cells from each group were suspended in 200 μl medium with 1 % FBS and placed on the Transwell inserts. The bottom chambers contained 600 μl of DMEM/ F12, 1 % FBS and 10 ng/ml SDF-1α. The plates were incubated at 37 °C for 24 h. Finally, the number of migrated cells was counted in 5 random fields. 

### Determining level of VEGFR-1, VEGFR-2, phosphorylated eNOS and eNOS in HUVECs treated with Crocetin

The levels of VEGFR-1, and -2, phosphorylated eNOS (p-eNOS) and eNOS were determined using enzyme-linked immune sorbent assay (ELISA) designed by our group. Briefly, we transferred 100 μl rabbit anti-human VEGFR-1 (Dilution: 1 µg/ml; Cat no: ab32152; Abcam), rabbit anti-human VEGFR-2 (Dilution: 1 µg/ml; Cat no: ab39256; Abcam), mouse anti-human eNOS (Cat no: sc-376542, Santa Cruz Biotechnology) and mouse anti-human phosphor eNOS (Cat no: sc-81510, Santa Cruz Biotechnology) into each well of polystyrene treated 96-well plate (SPL) and kept them overnight at 4 °C. The next day, the solution was discarded and the well surface was blocked by 1 % bovine serum albumin (Sigma). Thereafter, 100 µl protein lysate (dilution: 10 µg/ml) was added to each well and maintained at RT for 1 h. Following three times wash with PBS (each for 5 min), the wells were incubated with 100 µl anti-rabbit secondary HRP-conjugated antibodies (Dilution; 1: 3000; Cat no: ab6795; Abcam) for 45 min. Again, the wells were washed with PBS and 50 µl chromogenic substrate solution containing 3 % 3, 3', 5, 5'-tetramethylbenzidine (TMB) was added. To stop the reaction, 50 μl H_2_SO_4_ (5 %) was used and final absorbance was measured at 450 nm. The concentration of each factor was calculated based on comparison to a standard curve generated from samples of known VEGFR-1 and VEGFR-2. The level of both p-eNOS and eNOS was expressed as OD absorbance at 450 nm.

### Gelatin zymography

Metalloproteinase-2 and -9 (MMP-2 and -9) activities were studied by gelatin zymography. The cell lysate was prepared from each group using protein RIPA lysis buffer (Sigma) enriched with cocktail enzyme inhibitors (Sigma). The samples were electrophoresed by gelatin zymography (0.1 % gelatin; 10 % SDS-PAGE) under non-reducing conditions as previously reported by our group (Nemati et al., 2017[15]). After completion of electrophoresis, gels were washed twice with 2.5 % TritonX-100 buffer (each for 30 min) and incubated overnight at 37 °C in buffer containing 50 mM Tris-HCl, 200 mM NaCl, 10 mM CaCl_2_. For staining, we used 0.1 % Coomassie blue solution for 30 min. By using a solution containing 45 % methanol and 10 % acetic acid, the stained gels were destained. The intensity of clear band showing gelatinase activity of MMP-2 and -9 were calculated using ImageJ version 1.41.

### Lipoprotein lipase activity 

In normal conditions, ECs have the capacity to uptake low-density lipoprotein from circulation by lipoprotein lipase activity, which demonstrates the healthy status of these cells. To show the possible role of Crocetin on ECs LDL uptake, we performed Ac-LDL-Dil assay. 1 × 10^4 ^cells were plated in each well of Chamber Slide™ system 8 wells and treated with Crocetin as above-mentioned. Then, the media were discarded and 1 µg/ml acetylated-low density lipoprotein labeled with the fluorochrome Dil (Ac-LDL-Dil) was added to each well and kept for 4 h at 37°C. Cells were then washed twice with PBS and fixed with pre-cooled paraformaldehyde solution (4 %) for 15 min. For background staining, we used 1 µg/ml of DAPI (Sigma). The images were taken by fluorescence microscopy (Olympus, BX51) and the data was processed with CellSense software (version 1.12). This assay was performed in triplicate.

### Reverse transcription PCR (RT-PCR) 

To compare the transcription level of *VEGF* in Crocetin-treated cells with the control group, total RNA was extracted from each group using MATRIX universal RNA purification kit (Eurx, Poland). The content and quality of RNA were verified by a Picodrop spectrophotometer (Model: PICOPET01, UK). cDNA was synthesized using a cDNA synthesis kit (Cat no: k-2046, Bioneer). RT‐PCR was performed using specific primers for *VEGFA* and *β-actin* (used as an internal control) (Table 1(Tab. 1)). PCR cycling conditions for the genes were done as follows; an initial denaturation step at 95 °C for 10 min, followed by 45 amplification cycles consisting of denaturation at 95 °C for 15 sec, annealing at 60 °C for 30 sec and an extension at 72 °C for 30 sec. PCR products were electrophoresed on 1.5 % agarose gel. Gels were visualized and imaged by a Gel Doc image analyzer (Model: GBoxEF SYNGENE) and bands quantified by ImageJ software.

### Detection of phosphorylated Akt/Akt ratio by Western blotting 

The Western blotting analysis was performed to investigate a possible effect of Crocetin on the phosphorylation of Akt. To ascertain that Crocetin could promote angiogenic response via the Akt signaling pathway, we used PI3K inhibitor Wortmannin (100 nM; Sigma). Equal amounts of cell lysate (50 µg) were separated by SDS-PAGE and transferred to polyvinylidene difluoride membrane (Millipore). For the detection of target proteins, we used mouse anti-human Akt (Cat no: sc-56878, Santa Cruz Biotechnology) and mouse anti-human phosphor-Akt (Cat no: sc-81433, Santa Cruz Biotechnology). To detect immunoreactive bands ECL plus solution was used (Cat no: CMGECL). For densitometric analysis of immunoreactive bands, we used ImageJ software ver.1.44p (NIH). Results were expressed as relative density. For each test, we used β-actin as an internal control. This assay was performed in triplicate.

### Statistical analysis

All of the experiments were performed in triplicate. Quantitative data from the experiments were expressed as mean ± SD. Significance was determined by one-way analysis of ANOVA followed by Tukey's test. *p*<0.05 was considered statistically significant.

## Results

### Crocetin increased HUVECs viability and proliferation 

We found that HUVECs treatment with different doses of Crocetin induced cell proliferation over a period of 72 h (Figure 1B(Fig. 1)). WST-1 assay confirmed the effect of Crocetin in increasing HUVECs viability in a dose-dependent manner. Our results indicated that HUVECs treated with 50 µM Crocetin had the highest cell proliferation rate (Figure 1B(Fig. 1)). Compared to the control group, Crocetin had no effect on HUVECs proliferation rate after 24 hours while these effects reached the maximum levels after 72 hours. Noteworthy, lower concentrations of Crocetin (below 50 µM) contributed to near-normal values, especially during the first 24 h after treatment (Figure 1B(Fig. 1)). These data confirmed the effect of Crocetin in increasing HUVECs viability in a dose-dependent manner. Using 100 nM Wortmannin, an inhibitor of phosphoinositide 3-kinases (PI_3_K), we observed a significant decrease in Crocetin-induced cell viability (*p*<0.001; Figure 1C(Fig. 1)). Similarly, cell exposure to 1 µM N-monomethyl arginine (NMA; an eNOS inhibitor) contributed to the reduction of HUVECs survival rate compared to the control Crocetin after 72 h (*p<*0.001; Figure 1C(Fig. 1)). These data showed that the stimulatory effect of Crocetin on ECs proliferation is possibly mediated by PI_3_K/eNOS signaling pathway.

### Crocetin promoted in vitro tubulogenesis

**Figure 2:**
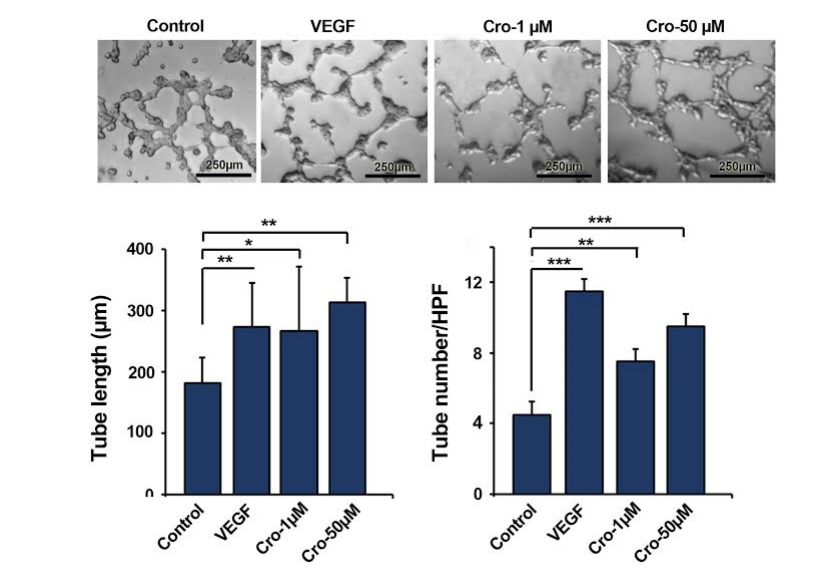
Representative image of *in vitro* tube formation assay. In HUVECs received 50 µM Crocetin, the highest tube length index was observed. Cell treatment with Crocetin increased both tube number and length values. Data are expressed as mean ± SD (n=3). *p<0.05, **p<0.01, ***p<0.001 using one-way ANOVA with Tukey post-hoc test.

Statistical analysis showed that Crocetin had a significant effect on the angiogenic potential of HUVECs (Figure 2(Fig. 2)). We found that 1 and 50 µM of Crocetin significantly increased tube length compared to the control HUVECs (Crocetin 1 µM Vs. Control: p<0.05; Crocetin 50 µM Vs. Control: p<0.01) (Figure 2(Fig. 2)). HUVECs given 50 µM Crocetin showed a higher tube length index than the cells treated with 1 µM Crocetin. The tube number in HUVECs exposed to 1 and 50 µM of Crocetin was also higher in comparison with the control cells (Crocetin 1 µM Vs. Control: p<0.01; Crocetin 50 µM Vs. Control: p<0.001). We found the highest tube number in VGEF-treated HUVECs as compared to other groups. These data demonstrated that Crocetin promoted the angiogenic potential of human ECs *in vitro* by stimulating vessel-like units.

### Crocetin contributed to an increased ECs migration rate

**Figure 3:**
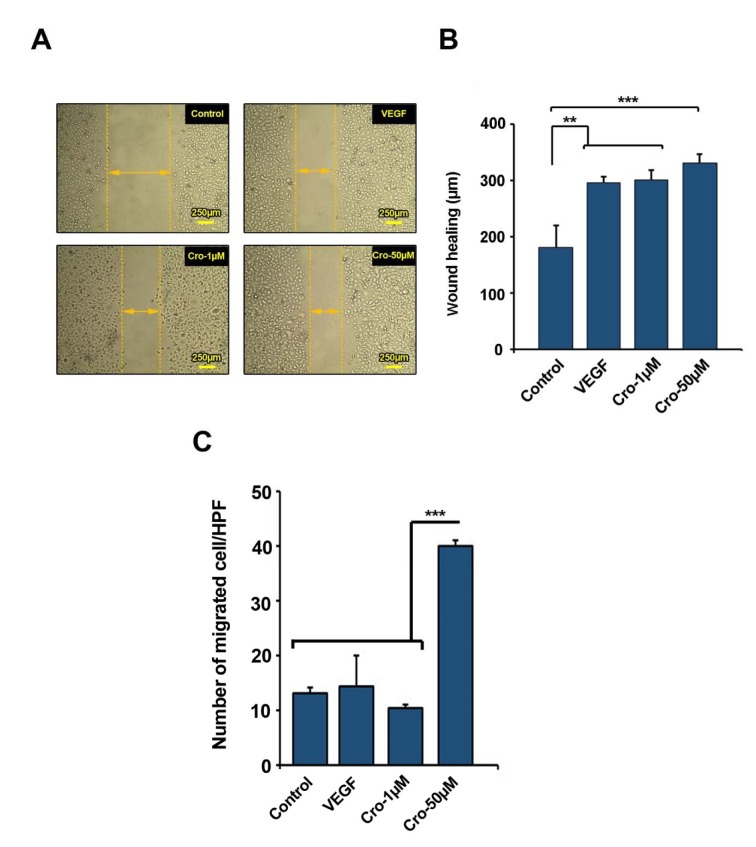
Scratch assay was performed to measure cell migration 72 h after being exposed to Crocetin (A-B) (n=3). Transwell migration assay (C) (n=3). Data are expressed as mean ± SD. *p<0.05, **p<0.01, ***p<0.001 using One-way ANOVA with Tukey post-hoc test.

We further monitored the stimulatory effect of Crocetin on HUVECs motility and migration rates (Figure 3A-C(Fig. 3)). For this purpose, *in vitro* scratch assay and Transwell inserts analysis was done (Figure 3A(Fig. 3)). Measuring the distance between edges indicated that Crocetin at 1 and 50 µM resulted in a significant cell movement rate as compared to control (Crocetin 1 µM Vs. Control: p<0.01; Crocetin 50 µM Vs. Control: p<0.001). We noted the same stimulatory effect in HUVECs incubated with 10 ng/ml VEGF (Figure 3A and 3B(Fig. 3)). We showed that HUVECs exposure to 50 µM Crocetin for 72 h significantly increased the number of migrated cells through an 8-μm pore size membrane as compared to control or HUVECs from other groups (Figure 3C(Fig. 3)). The number of migrated cells correlated with an increasing dose of Crocetin *in vitro*. Compared to the cells receiving 1 µM Crocetin or 10 ng/ml VEGF, 50 µM Crocetin had a superior effect in promoting HUVECs migration, wherein a ~2.5-fold increase in cell migration was observed in comparison with the control cells (*p*<0.001). These data demonstrated that Crocetin increased ECs migration in proportion to the increasing doses of Crocetin *in vitro*.

### Protein level of VEGFR was elevated in Crocetin-treated ECs 

**Figure 4:**
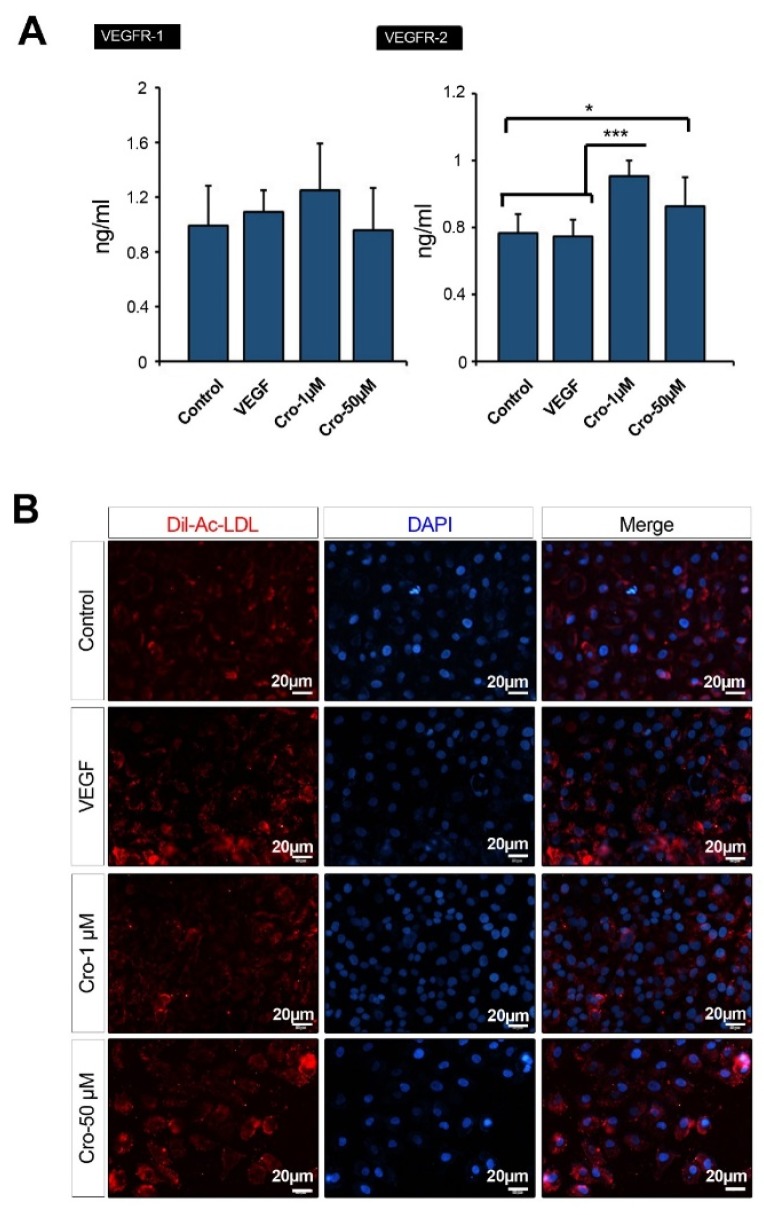
Measuring the cellular content of VEGFR-1 and VEGFR-2 by ELISA (A) (n=8) by using One-way ANOVA and Tukey's Post Hoc. *p<0.05; ***p<0.001. Representative image of lipoprotein lipase activity evaluated by Ac-LDL-Dil uptake assay)B( (*n*=3).

VEGF-R1 and VEGFR-2 are certain Receptor Tyrosine Kinases (RTKs) to transmit VEGF effects, contributing to a pro-angiogenic switch inside endothelial lineage. To address the impact of Crocetin on the protein content of VEGF-R1 and -2, we performed an ELISA assay (Figure 4A(Fig. 4)). ELISA analysis of VEGFR-1 and -2 in HUVECs incubated with Crocetin showed an elevated protein content of VEGFR-2 as compared to non-treated control cells (Control Vs. Crocetin 50 µM: *p*<0.05; Control Vs. Crocetin 1 µM: *p* <0.001 and VEGF Vs. Crocetin 1 µM: *p*<0.001). The concentration of VEGFR-1 similarly increased in HUVECs treated with 1 and 50 µM Crocetin, but it did not reach a significant level (*p*>0.05). Noteworthy, we found the highest levels of VEGFR-1 and VEGFR-2 proteins in HUVECs receiving 1 µM Crocetin (Figure 4A(Fig. 4)). The increase of both VEGFR-1 and VEGFR-2 in HUVECs showed a pro-angiogenic activity of Crocetin in ECs by modulating the kinetic of RTKs.

### AC-LDL-Dil uptake was increased after cell treatment with Crocetin 

Using Ac-LDL-Dil uptake analysis, we monitored lipoprotein lipase activity in ECs exposed to Crocetin for 72 h (Figure 4B(Fig. 4)). We found that HUVECs exposed to 50 µM Crocetin, but not 1 µM Crocetin, were able to efficiently uptake Ac-LDL. After 72 h of HUVECs incubation with 50 µM Crocetin, the fluorescent intensity was a similar magnitude in comparison to VEGF-treated cells. A lower activity of lipoprotein lipase activity and near-to-control intensity was indicated in HUVECs given 1 µM Crocetin (Figure 4B(Fig. 4)). These data confirmed that ECs exposure to Crocetin improved functional lipoprotein lipase activity after 72 h, playing a crucial role in the normal activity of endothelial lineage in response to lipids.

### Crocetin enhanced MMP activity in HUVECs

**Figure 5:**
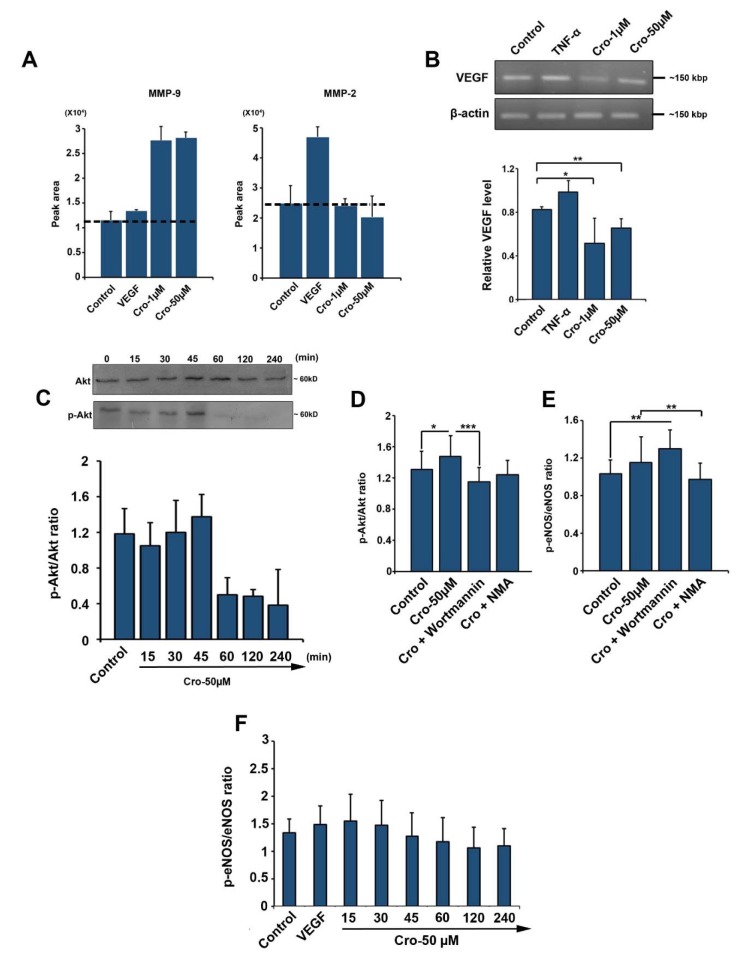
Gelatin zymogram showing MMP-9 and MMP-2 activities in HUVECs treated with Crocetin (A) (n=3). RT-PCR analysis of VEGF expression (B) (n=3). Western blot analysis of p-Akt/Akt ratio (C). Western blotting revealed the non-significant increase of p-Akt/Akt ratio during the 45 min-incubation of HUVECs with 50 µM Crocetin and these effects were diminished from time points 60 to 120 min. (n=3). Measuring the p-Akt/Akt and p-eNOS/eNOS ratios by ELISA (D, E, and F) (n=12) by using One-way ANOVA and Tukey's Post Hoc. *p<0.05; **p<0.01; ***p<0.001.

According to data from gelatinase zymography analysis, we found an increase in MMP-9 activity in HUVECs exposed to 1 and 50 µM Crocetin (Figure 5A(Fig. 5)). The analysis of protein lysates from different groups showed that Crocetin, at both concentrations, was not able to increase MMP-2 activity after 72 h (Figure 5A(Fig. 5)). In line with these results, one could hypothesize that Crocetin is able to enhance the angiogenesis potential of ECs by activating distinct types of MMPs.

### Expression of VEGF was decreased in the presence of Crocetin 

Relative expression of the VEGF gene was detected using RT-PCR analysis. We found that the transcription of VEGF was reduced in HUVECs given 1 and 50 µM Crocetin (Control Vs. Crocetin 1 µM: *p*<0.05 and Control vs. Crocetin 50 µM: *p*<0.01; Figure 5B(Fig. 5)). As expected, the incubation of HUVECs with TNF-α increased the expression of VEGF (Figure 5B(Fig. 5)). These data showed that total VEGF mRNA content was decreased in Crocetin-treated cells which may possibly link to a reduction in VEGF expression or increase in translation rate and protein synthesis. 

### Crocetin modulated the p-Akt/Akt and p-eNOS/eNOS ratios 

The analysis of protein lysate from HUVECs incubated with 50 µM Crocetin revealed an increase in p-Akt/Akt ratio with a maximum level during the first 45 min treatment and the results were not statistically significant (p>0.05; Figure 5C(Fig. 5)). We found that p-Akt/Akt ratio started to decrease 60 minutes upon treatment with Crocetin and lowest levels were observed at minute 240. The blocking of PI3K by Wortmannin decreased p-Akt/Akt ratio and returned it to levels below the control values (Crocetin VS. Crocetin + Wortmannin: *p* <0.001; Control VS. Crocetin: *p* <0.05; Figure 5D(Fig. 5)). Despite the decline in p-Akt/Akt ratio of Wortmannin-treated HUVECs, no significant changes were observed compared to non-treated control cells (*p*>0.05; Figure 5D(Fig. 5)). Furthermore, upon treatment of HUVECs with Crocetin, the production of p-eNOS was increased and p-eNOS/eNOS ratio reached to highest level during the first 15 min (Figure 5E-5F(Fig. 5)). All changes were statistically non-significant (p>0.05). Thirty minutes after HUVECs exposure to Crocetin, this ratio decreased and reached the lowest rate during the first 240 minutes. Blocking eNOS by NMA decreased the Crocetin-increased p-eNOS/eNOS ratio as compared to HUVECs receiving only Crocetin (Crocetin vs. Crocetin + NMA: *p* <0.01; Figure 5E(Fig. 5)). Commensurate with these results, it seems reasonable to suggest that Crocetin affect HUVECs by the modulation of p-Akt/Akt and p-eNOS/eNOS ratios.

## Discussion

Promoting angiogenesis seems to be an appropriate strategy for the alleviation of ischemic changes and healing of numerous tissues. Numerous attempts have been made to find phyto-compounds promoting angiogenesis and vasculogenesis (Hassanpour et al., 2016[11]). In the current experiment, we investigated the pro-angiogenic effect of Crocetin on human ECs *in vitro*. The results of our experiment showed that HUVECs survival rate increased in a dose-dependent manner after 72 hours compared with that of the control group. Consistent with our results, many authors showed the stimulatory effect of Crocetin on different cell types (Ghorbanzadeh et al., 2017[10]; Yang et al., 2018[23]). It has been shown that Crocetin can provoke different biological mechanisms, resulting in an increased cell viability rate (Meng and Cui, 2008[14]; Song et al., 2016[18]; Yamauchi et al., 2011[22]). For instance, Crocin, an analog of Crocetin, inhibits the apoptosis rate in human ECs induced by trisol (Meng and Cui, 2008[14]). Crocetin reduces the content of intracellular reactive oxygen species (ROS) and stabilizes calcium ion in bovine aortic ECs exposed to oxidized low-density lipoprotein and advanced glycation end products (Wang et al., 2019[20]; Xiang et al., 2006[21]). The application of Crocetin improved the survival rate after bacterial lipopolysaccharide-induced acute lung injury by down-regulating the NF-ĸB signaling pathway (Yang et al., 2012[24]). As a matter of fact, the inhibition of ROS generation, pro-inflammatory response and apoptosis can increase the cell survival rate (Sart et al., 2015[17]).

Moreover, we noted that Crocetin enhanced the HUVECs tubulogenesis and formation of capillary-like structure *in vitro* along with the increase of migration. These features are related to pro-angiogenesis status. In line with these results, Ghorbanzadeh and co-workers showed an increase angiogenesis rate in cardiac tissue by up-regulating miR-126, miR-210 expression, and CD31 after Crocetin use (Ghorbanzadeh et al., 2017[10]). Administration of saffron containing Crocetin also enhanced the angiogenesis rate in rats' brain following experimental stroke (Bie et al., 2011[2]). Compared to the control group, an increased lipoprotein lipase activity was found upon cell treatment with Crocetin, showing the improvement of lipid metabolism in ECs.

By exposing HUVECs to Crocetin, levels of VEGFR-1 and VEGFR-2 also increased. These receptors are involved in the angiogenesis mediated by VEGF signaling pathway (Rahimi, 2006[16]). Therefore, the use of Crocetin supports the functional acquisition of human ECs mediated by induction of VEGFR-1 and VEGFR-2 (Bie et al., 2011[2]). Consistent with the increase of HUVECs migration, a greater MMP-9 activity, but not MMP-2, was observed in cells treated with Crocetin. Previously, it was found that VEGFRs activation initiated cell migration via the regulation of MMP-9 activity (Sart et al., 2015[17]). Treatment of breast cancer cell line with Crocetin reduced metastasis rate by suppressing MMP-2 activity and inhibition of pro-MMP-9 conversion to MMP-9 (Chryssanthi et al., 2011[4]). Such differences in the activity of MMP-2 and MMP-9 in the current experiment are possibly due to the distinct Crocetin concentration and incubation time. 

Under our experimental condition, VEGF expression by ECs was down-regulated. One explanation would be that the reduction of VEGF mRNA was a compensatory response to an induced VEGFRs activity. 

We also confirmed that p-eNOS/eNOS and p-Akt/Akt ratios were induced in the presence of Crocetin. It was previously reported the induction of RTKs such as VEGFR-1 and VEGFR-2 initiated downstream signals via applying the PI3K/Akt/eNOS axis (Feliers et al., 2005[7]). Consistent with our data, Meng and Cui (2008[14]) revealed the activation of PI3K/Akt/eNOS pathway after treatment with Crocetin. They also reported that the promotion of PI3K/Akt/eNOS decreased high glucose content-induced cytotoxicity in ECs following treatment with Crocetin. By blocking the activity of eNOS and PI3K via commercial inhibitors such as Wortmannin and NMA, Crocetin effect on ECs was observed. These features indicate that Crocetin exerts the therapeutic effect on HUVECs by evoking PI3K/Akt/eNOS signaling pathway and VEGFR-1 and VEGR-2.

## Conclusion

In summary, the current experiment supports the notion that Crocetin improves angiogenesis potential of human ECs by improving the migration and cell survival rate. The cell distribution of RTKs receptor increase coincided with the activation of the PI3K/Akt/eNOS axis. These results shed lights on the beneficial effects of Crocetin in promoting angiogenesis in *in vitro* and *in vivo* conditions. Further investigations are required to elucidate the precise underlying mechanism after cell exposure to Crocetin. 

## Acknowledgement

The authors appreciate the personnel of Stem Cell Research Center of Tabriz University of Medical Sciences for guidance during all phases of the experiment.

## Conflict of interest

There is no conflict of interest to declare.

## Figures and Tables

**Table 1:**
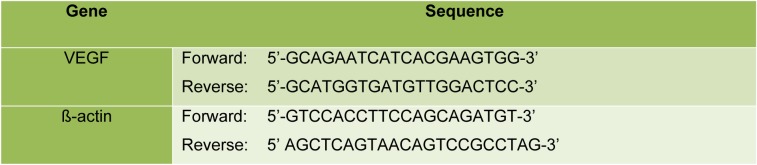
Primer list
